# Association of Heart Rate Variability in Taxi Drivers with Marked Changes in Particulate Air Pollution in Beijing in 2008

**DOI:** 10.1289/ehp.0900818

**Published:** 2009-09-16

**Authors:** Shaowei Wu, Furong Deng, Jie Niu, Qinsheng Huang, Youcheng Liu, Xinbiao Guo

**Affiliations:** 1 Peking University School of Public Health, Beijing, China;; 2 Peking University Third Hospital, Beijing, China;; 3 University of Kentucky College of Public Health, Lexington, Kentucky, USA

**Keywords:** air pollution, cardiac autonomic function, epidemiology, heart rate variability, particulate matter

## Abstract

**Background:**

Heart rate variability (HRV), a marker of cardiac autonomic function, has been associated with particulate matter (PM) air pollution, especially in older patients and those with cardiovascular diseases. However, the effect of PM exposure on cardiac autonomic function in young, healthy adults has received less attention.

**Objectives:**

We evaluated the relationship between exposure to traffic-related PM with an aerodynamic diameter ≤ 2.5 μm (PM_2.5_) and HRV in a highly exposed panel of taxi drivers.

**Methods:**

Continuous measurements of personal exposure to PM_2.5_ and ambulatory electrocardiogram monitoring were conducted on 11 young healthy taxi drivers for a 12-hr work shift during their work time (0900–2100 hr) before, during, and after the Beijing 2008 Olympic Games. Mixed-effects regression models were used to estimate associations between PM_2.5_ exposure and percent changes in 5-min HRV indices after combining data from the three time periods and controlling for potentially confounding variables.

**Results:**

Personal exposures of taxi drivers to PM_2.5_ changed markedly across the three time periods. The standard deviation of normal-to-normal (SDNN) intervals decreased by 2.2% [95% confidence interval (CI), −3.8% to −0.6%] with an interquartile range (IQR; 69.5 μg/m^3^) increase in the 30-min PM_2.5_ moving average, whereas the low-frequency and high-frequency powers decreased by 4.2% (95% CI, −9.0% to 0.8%) and 6.2% (95% CI, −10.7% to −1.5%), respectively, in association with an IQR increase in the 2-hr PM_2.5_ moving average.

**Conclusions:**

Marked changes in traffic-related PM_2.5_ exposure were associated with altered cardiac autonomic function in young healthy adults.

Many studies have linked both short-term and long-term ambient particulate matter (PM) air pollution to increased morbidity and mortality of cardiovascular diseases in the general population (Clancy et al. 2002; Dockery 2001; Goldberg et al. 2006; Larrieu et al. 2007), but the biologic mechanisms of these associations remain unclear. Altered cardiac autonomic function, as reflected by alterations of heart rate variability (HRV), is considered one of the pathophysiologic pathways through which PM air pollution influences the cardiovascular system (Pope et al. 2004a; Rhoden et al. 2005). Increased PM air pollution has been associated with declines in HRV in older people and in patients with current or underlying cardiovascular diseases (Gold et al. 2000; Liao et al. 1999; Park et al. 2005; Pope et al. 2004b; Riojas-Rodriguez et al. 2006; Schwartz et al. 2005), suggesting that they are susceptible to the ambient PM air pollution. On the other hand, results from previous studies on the relationship between PM exposure and HRV in younger individuals have not been consistent. Some studies have observed negative exposure–response associations between PM exposure and HRV (Cavallari et al. 2007; Magari et al. 2001, 2002a; Vallejo et al. 2006), whereas others have report positive associations (Magari et al. 2002b; Peretz et al. 2008; Riediker et al. 2004). These different findings suggest a complicated mechanism through which the cardiac autonomic system responds to PM exposure in younger individuals.

The ambient PM air pollution in Beijing during the 2008 Olympic Games (from 8 August to 20 September) decreased markedly after a series of air quality control measures were implemented by the Beijing municipal government, including banning the use of more than half of the motor vehicles in Beijing every day. The marked changes of PM air pollution before, during, and after the Beijing 2008 Olympic Games provided a unique opportunity to investigate the effect of a wide range of PM exposure on cardiac autonomic function. We conducted an occupational panel study to evaluate the relationship between the PM_2.5_ exposure and changes in HRV in a group of young, healthy taxi drivers who were exposed to relatively high traffic-related PM air pollution before, during, and after the Beijing 2008 Olympic Games. The objectives of this study were *a*) to determine whether reduced PM air pollution in Beijing during the Olympic Games improved the HRV, and *b*) to quantitatively evaluate the percent changes of HRV corresponding to the increase of PM levels by controlling potentially confounding variables.

## Materials and Methods

### Study subjects and protocol

This study was designed as a panel study. Forty-four young, nonsmoking taxi drivers were recruited from several taxi companies in Beijing. A self-administered questionnaire was used to collect personal information, including name, sex, age, years of employment as a taxi driver, smoking status, education, and history of cardiovascular diseases or other diseases. Drivers were then physically examined and tested for seated blood pressure, resting electrocardiogram (ECG), blood cholesterol, triglycerides, and high- and low-density lipoproteins. To increase the homogeneity of subjects and reduce confounding, we used the following inclusion criteria to select subjects for this analysis: nonsmoking; no history of physician-diagnosed cardiovascular, pulmonary, neurologic, or endocrine diseases; body mass index (BMI) ≤ 30, normal blood pressure; normal resting ECG and normal blood test results; daytime taxicab driving hours; and employment as a taxi driver for at least 1 year. Using these criteria, a total of 14 taxi drivers were initially selected to participate in the study. The study was approved by the Institutional Review Board of Peking University Health Science Center, and informed consent was obtained from each subject before the study began.

Continuous measurements of personal exposure to PM with an aerodynamic diameter ≤ 2.5 μm (PM_2.5_) and ambulatory ECG monitoring were conducted on each subject during a 12-hr work shift (0900–2100 hour) before (26 May to 19 June), during (11 August to 5 September), and after (27 October to 14 November) the Beijing 2008 Olympic Games. Measurements were performed only on weekdays (from Monday to Friday) to reduce variation due to changes in traffic flow across the week (weekend traffic was more irregular).

### HRV measurements

A three-channel Holter recorder (model MGY-H7; DM Software Inc., Stateline, NV, USA) was used for ambulatory ECG monitoring. Each subject’s skin was carefully cleaned with alcohol pads by a trained technician before the electrodes were connected. Modified V_1_ and V_5_ leads were used to obtain the cardiac electrophysiologic signals. Each subject wore the Holter recorder for 12 hr from the beginning (0900 hour) to the end (2100 hour) of the work shift, and each subject was also given a daily work diary to record personal activities such as eating or resting. Each 12-hr ambulatory ECG tape was processed using the software for the Holter recorder (Holter System, version 12.net; DM Software Inc.). Only normal-to-normal (NN) beat intervals from 600 msec to 1,500 msec with NN ratios between 0.8 and 1.2 were included in the data analysis. All abnormities and noises were excluded based on standard criteria (Task Force of the European Society of Cardiology and the North American Society of Pacing and Electrophysiology 1996) to ensure the quality of data. Three indices of HRV were calculated and used in the analysis: *a*) standard deviation of NN intervals (SDNN), *b*) low-frequency (LF) power (0.04–0.15 Hz), and *c*) high-frequency (HF) power (0.15–0.40 Hz). These HRV indices were all calculated in standard 5-min segments throughout the entire recording, but data collected when drivers were not in the taxicab were deleted to reduce confounding by nondriving-related activities. Because the subjects spent most of their work time inside the taxicab and always kept a stationary sitting posture, the monitoring environment was considered relatively stable. Consequently, 5-min HRV indices could be used to analyze the association between personal PM exposure and HRV with little interference due to changes in activity level or position.

### Exposure to air pollutants and microclimate variables

Air pollutant concentrations were measured continuously and simultaneously using monitoring equipment installed inside each taxicab. Real-time concentrations of PM_2.5_ were measured by a portable aerosol spectrometer (model 1.109; Grimm Technologies Inc., Douglasville, GA, USA), logged in 1-min intervals, and aggregated as 5-min averages. The daily mean concentration of PM_2.5_ for each driver was obtained directly from the equipment by averaging all data points obtained during the day. PM_2.5_ mass was also collected gravimetrically on polytetrafluoroethylene filters (Whatman Inc., Florham Park, NJ, USA) using an SKC sampling system (2.5-μm personal environmental monitor and AirChek XR5000 sampling pump; SKC Inc., Eighty Four, PA, USA). Real-time measurements of PM_2.5_ were influenced by seasonal changes in weather. Therefore, real-time concentrations were calibrated based on the average PM_2.5_ mass concentration obtained from the gravimetric analysis each day to ensure a more reliable measure of individual exposure [see Supplemental Material available online (doi:10.1289/ehp.0900818.S1 via http://dx.doi.org/)].

Concentrations of carbon monoxide (CO), nitrogen dioxide (NO_2_), and nitric oxide (NO) were also measured in order to evaluate confounding effects of these gaseous pollutants on the relationship between PM_2.5_ and HRV. CO was measured with a model T15n enhanced CO measurer (Langan Products Inc., San Francisco, CA, USA), and NO_2_ and NO were measured using a passive sampler (Ogawa Air Inc., Osaka, Japan) and analyzed according to the manufacturer’s specifications. Real-time temperature and relative humidity (RH) data were measured using a HOBO Pro V2 temperature/RH logger (Onset Corp., Pocasset, MA, USA), logged at 1-min intervals and aggregated as 5-min averages.

Traffic routes were recorded using a model M-241 wireless global positioning system (GPS) logger (HOLUX Technology Inc., Hsinchu, Taiwan). Drivers used a work diary to record their activities, car window status (open vs. closed), and air conditioner use (on vs. off) throughout the monitoring periods.

### Statistical analysis

Field data were checked for completeness on site, and efforts were made to ascertain missing data as needed. HRV indices were log_10_-transformed to improve normality and stabilize the variance. Data collected when drivers were not in their taxicab were identified based on work diaries and GPS records and were excluded before analysis. All statistical analyses were performed using SAS software for Windows (version 9.1; SAS Institute Inc., Cary, NC, USA).

We conducted measurements on each subject during each time period in order to compare associations between different PM_2.5_ exposure levels and HRV. To facilitate logical and accurate comparisons and interpretation, we tested the data structure for each subject before analysis. The number of 5-min HRV indices for each subject in each of the three time periods should have been approximately equal; otherwise, the distribution of raw 5-min HRV indices might have been biased. We excluded 3 subjects from the final analysis who had substantially fewer observations in one of the three time periods relative to the other two because of less time (< 5 hr) spent inside the taxicab during that shift. Therefore, data from the remaining 11 subjects were used for the analysis on the association between PM_2.5_ exposure and HRV.

We first compared raw 5-min HRV indices during the three time periods using one-way analysis of variance (ANOVA) and then used mixed-effects regression models to analyze combined data from all three time periods and estimate associations of varying exposure levels of PM_2.5_ with HRV. Subject and day of the year were treated as random effects, whereas age, time of day, log_10_-transformed heart rate, temperature, and RH were included as fixed effects. The gaseous pollutants CO, NO_2_, and NO were also individually evaluated as copollutants in two-pollutant models with PM_2.5_. Both linear and quadratic terms were used to model moving averages of temperature and RH to account for nonlinear associations with HRV. Other potential confounders were also investigated: sex, BMI, years as a taxi driver, day of week, status of cab windows, and status of cab air conditioner. These variables did not show significant confounding effects in any of the regression models. Therefore, final models included random effects terms for subject and day of the year and fixed effects terms for age, time of day, log_10_-transformed heart rate, temperature, and RH only.

Seven different PM_2.5_ moving averages were generated for lagged intervals ranging from 5 min to 4 hr using the calibrated 5-min average real-time PM_2.5_ concentrations. Corresponding log_10_-transformed 5-min HRV indices were regressed on the different PM_2.5_ moving averages. The 30-min and 2-hr moving averages were chosen as the main exposure metrics because PM_2.5_ effects on HRV were mainly observed for these two lagged intervals. Analyses examining the influence of outlying exposure values on regression results were also performed after excluding observations in the top 5% and bottom 5% of the PM_2.5_ moving averages, respectively.

Final results are presented as the estimated percent change in each 5-min HRV index associated with an interquartile range (IQR) increase of each air pollutant as


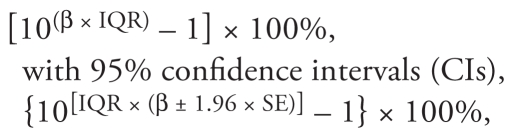


where β and SE are the effect estimate and its standard error.

## Results

Table 1 presents detailed information on the characteristics of the 11 subjects included in the final analysis. Six were females (55%), the mean age of all subjects was 35.5 years (range, 27–41 years), and the mean time of employment as a taxi driver was 6.0 years. All 11 subjects were in good health with blood pressures, resting heart rate, and blood cholesterol, triglycerides, and lipoproteins in normal ranges. None of the subjects had a history of cardiovascular diseases or smoking.

In Table 2, we show the daily averages of air pollutant levels and microclimate variables inside the taxicab during each of the three time periods. According to the diaries filled in by drivers, they spent an average of 87% of their work time inside the taxicab each day; thus, the results of exposure monitoring represent the taxi drivers’ typical personal exposure levels during their work time. PM_2.5_ exposure levels are presented as daily averaged real-time PM_2.5_ concentrations and daily averaged PM_2.5_ mass concentrations obtained from gravimetric analysis. Results from the two methods are highly correlated (*r* = 0.973, *p* < 0.001); therefore, we present results for PM_2.5_ mass concentrations unless otherwise indicated. Average individual air pollutant exposure levels during the Olympic Games were much lower than levels before and after the Olympic Games, especially levels of PM_2.5_, which decreased to less than half the average level recorded before the Olympic Games (45.2 μg/m^3^ vs. 105.5 μg/m^3^, respectively). The study lasted for about half a year, from the end of spring to the end of fall, resulting in differences in temperature and RH across the three time periods.

Table 3 presents the distribution of raw 5-min HRV indices across the three time periods. It is evident that all three indices of HRV were higher during the Olympic Games than before and after the Olympic Games. One-way ANOVA indicated significant differences for all three 5-min HRV indices among the three time periods (*p* < 0.0001).

**Figure 1:**
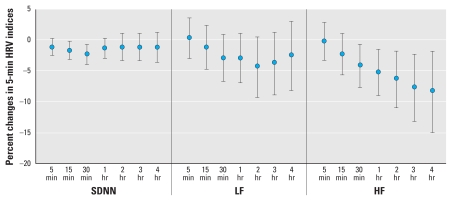
Percent changes (95% CIs) in 5-min HRV indices associated with an IQR (69.5 μg/m^3^) increase of the PM_2.5_ for moving averages from 5 min to 4 hr.

We estimated associations between the 5-min HRV indices and different PM_2.5_ moving averages (from 5 min to 4 hr) after combining data for each subject across the three time periods and adjusting for potentially confounding variables. IQR increases in PM_2.5_ mass concentrations were associated with declines in all three 5-min HRV indices (Figure 1). Table 4 shows adjusted associations between IQR increases in 30-min and 2-hr PM_2.5_ moving averages and the 5-min HRV indices. An IQR (69.5 μg/m^3^) increase in the 30-min PM_2.5_ moving average was associated with a 2.2% decline (95% CI, −3.8% to −0.6%) in the SDNN. Associations with LF and HF powers were stronger for an IQR increase in the 2-hr PM_2.5_ moving average, with decreases of 4.2% (95% CI, −9.0% to 0.8%) and 6.2% (95% CI, −10.7% to −1.5%), respectively. We also performed the same regression models after excluding observations in the top 5% and bottom 5% of the PM_2.5_ moving averages, to evaluate the influence of outlying exposure values on associations. Effect estimates became more negative, especially those for LF and HF powers in association with the 30-min PM_2.5_ moving average.

**Figure 2:**
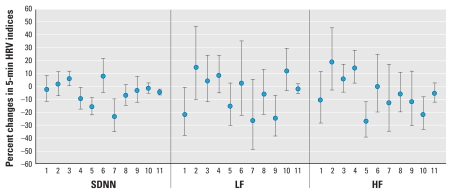
Subject-specific effect estimates (percent changes with 95% CIs) for 5-min HRV indices associated with an IQR (69.5 μg/m^3^) increase in the 30-min PM_2.5_ moving average. The *x*-axis indicates different subjects (subjects 1–6 are females; subjects 7–11 are males).

To further explore potential heterogeneity from person to person, we fitted subject-specific regression models using data from all three time periods. Figure 2 shows the distribution of the subject-specific effect estimates for an IQR increase in the 30-min PM_2.5_ moving average for all three 5-min HRV indices. The relatively wide range of effect estimates suggests that individual heterogeneity was present despite the strict inclusion criteria used to select participants for the study. For example, 8 effect estimates for percent change in SDNN with an IQR increase in PM_2.5_ were negative, and 3 were positive. Results for LF and HF powers were similar.

**Figure 3:**
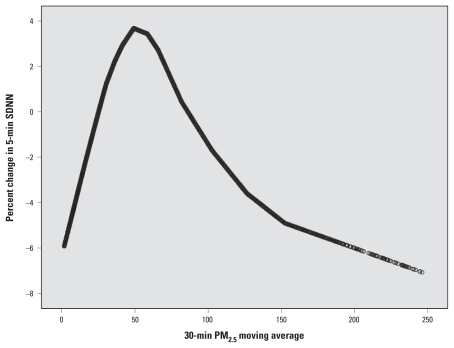
Smoothed curve showing percentage deviation from mean predicted 5-min SDNN according to the 30-min PM_2.5_ moving average (based on the model with all other covariates adjusted). The 30-min PM_2.5_ moving average of the turning point for opposite association is 49.3 μg/m^3^.

Furthermore, we fitted exposure–response curves for PM_2.5_ on 5-min HRV indices based on regression models combining data across the three time periods. Figure 3 shows the estimated relationship between the 30-min PM_2.5_ and 5-min SDNN using a loess smoother with a smoothing parameter of 0.6. The relationship between PM_2.5_ and SDNN is not linear. Specifically, there is a positive association between PM_2.5_ and percent change in SDNN when PM_2.5_ concentrations are relatively low (up to 49.3 μg/m^3^), but the association becomes negative when PM_2.5_ concentrations are higher, and eventually levels off for the highest levels of PM_2.5_. Results for the LF and HF powers were similar to those for SDNN [see Supplemental Material, Figure 1 (doi:10.1289/ehp.0900818.S1)]. These findings may provide a reasonable explanation for the more negative estimated percent changes in 5-min HRV indices after excluding observations in the top 5% and bottom 5% of the PM_2.5_ moving averages (Table 4).

We investigated potential confounding by the gaseous pollutants CO, NO_2_, and NO by including each of them in a two-pollutant model with PM_2.5_. In general, adjusting for CO weakened effect estimates for PM_2.5_ on HRV, whereas adjusting for NO_2_ made them somewhat stronger. Adjusting for the copollutants decreased the precision of the estimates, but the overall results were generally consistent with estimates that were not adjusted for the copollutants [see Supplemental Material, Table 1 (doi:10.1289/ehp.0900818.S1)]. Separate models to estimate associations of CO, NO_2_, and NO with HRV generally did not support associations between these pollutants and the outcomes [see Supplemental Material, Table 2 (doi:10.1289/ehp.0900818.S1)].

## Discussion

In this study, we assessed real-time personal exposure to traffic-related PM_2.5_ across three time periods associated with substantial differences in exposure levels, and evaluated the relationship between PM_2.5_ and HRV as measured by ambulatory ECG in a selected group of young, healthy, nonsmoking taxi drivers. The levels of traffic-related PM_2.5_ to which taxi drivers were exposed in our study (especially the levels before the Olympic Games) were much higher than ambient or personal PM exposure levels reported in previous studies of populations in other countries (Holguín et al. 2003; Park et al. 2005; Riediker et al. 2004; Riojas-Rodriguez et al. 2006). To the best of our knowledge, this is the first study to show effects of varying levels of traffic-related PM exposure on cardiac autonomic function in a highly exposed occupational panel.

A major strength of this study is that we evaluated the same workers during three different time periods that had markedly different PM air pollution levels, which allowed us to compare the corresponding levels of 5-min HRV indices among different time periods. A comparison of raw 5-min HRV indices indicated that the low PM_2.5_ exposure period (during the Olympic Games) was associated with relatively high HRV, whereas higher PM_2.5_ exposures (before and after the Olympic Games) were associated with relatively low HRV.

Decreased HRV has been regarded as a risk factor for cardiac morbidity and mortality (La Rovere et al. 2003; Lombardi et al. 2001), and previous studies have linked decreased HRV to ambient PM air pollution (Gold et al. 2000; Liao et al. 1999, 2004; Magari et al. 2002a; Park et al. 2005; Pope et al. 2004b), occupational PM exposure (Cavallari et al. 2007; Magari et al. 2001, 2002a), and PM air pollution related to traffic (Schwartz et al. 2005). Our results suggest that marked changes of PM air pollution may lead to cardiac autonomic imbalance in young healthy individuals, as indicated by declines in several 5-min HRV indices in taxi drivers.

Several previous studies also have associated PM exposure with HRV in younger individuals (Cavallari et al. 2007; Magari et al. 2001, 2002a, 2002b; Peretz et al. 2008; Riediker et al. 2004; Vallejo et al. 2006), but results reported in these studies were not consistent with each other. A previous study of nine young, healthy, nonsmoking, male patrol troopers with a mean age of 27.3 years used short-term HRV (10 min) to evaluate the effect of traffic-related PM exposure on cardiac autonomic function (Riediker et al. 2004). The PM_2.5_ exposure levels in patrol cars were monitored for 4 consecutive days on each trooper during their work shift (from 1500 hr to midnight shift), and several periods of short-term HRV were assessed. Results showed that each 10-μg/m^3^ increase of in-vehicle PM_2.5_ (average of 24 μg/m^3^) was associated with a significant 11.7% increase in SDNN and a significant 14.8% increase in HF power the next morning. These findings suggest that that young, healthy individuals may respond to PM_2.5_ exposure differently than do older people and patients who have reduced cardiovascular dynamism and altered vagal (parasympathetic) function. On the other hand, some studies have reported declines of HRV in younger individuals who were exposed to PM occupationally (Cavallari et al. 2007; Magari et al. 2001) or environmentally (Magari et al. 2002a; Vallejo et al. 2006) at higher levels. Magari et al. (2001) observed a significant 2.66% (95% CI, −3.75% to −1.58%) decline in 5-min SDNN for every 1-mg/m^3^ increase in the 4-hr PM_2.5_ moving average due to occupational, personal PM_2.5_ exposure (arithmetic mean ± SD, 0.697 ± 0.665 mg/m^3^) in 33 boilermakers with a mean age of 38.1 years. Cavallari et al. (2007) found similar results in a group of male boilermaker welders who were exposed to an average PM_2.5_ level of 0.73 ± 0.50 mg/m^3^, using several indices of nighttime HRV (0000–0700 hr) as indicators; a 1-mg/m^3^ increase of 8-hr time-weighted average concentration of workday PM_2.5_ exposure was associated with a significant decline of 8.32 msec (95% CI, −16.29 to −0.35 msec) in nighttime sqare root of the mean squared differences of successive intervals (rMSSD), after adjusting for nonworking nighttime HRV, age, and smoking.

The results of our study are generally consistent with those that have reported declines of HRV with PM exposure in younger individuals. However, results from regression models for each subject showed heterogeneity among subjects, and we found that several subjects in our study had positive associations for the three 5-min HRV indices with traffic-related PM exposure. These subjects might have responded to PM in a similar way as reported by Riediker et al. (2004). On the other hand, smoothed curves showing associations between PM exposure and 5-min HRV indices also indicated that lower PM exposures were associated with increases in HRV whereas higher PM exposures were associated with decreases in HRV. Thus, differences between studies may also be related to differences in exposure levels. Overall, factors affecting heterogeneity of responses to PM exposure remain unclear, and further study is needed to explore the mechanisms behind the different responses to PM exposure in younger individuals.

Traffic density was different across the three time periods because of the Olympic Games (half of the motor vehicles in Beijing were banned from the streets each day during the Olympic Games), and this change in traffic density might have introduced a confounding effect by influencing the stress level of subjects, because previous studies have found associations between HRV and work-related stress (Kang et al. 2004; Saito et al. 2008; Vrijkotte et al. 2000). To partly control variation in stress levels across different time periods, we used a random effect for day of the year in regression models.

In summary, our study revealed an association between PM exposure and HRV in a group of young, healthy taxi drivers who were exposed to much higher traffic-related PM air pollution than the general population. This finding provides further evidence that PM air pollution affects cardiac autonomic function in young, healthy individuals.

## Figures and Tables

**Table 1:** Characteristics of study subjects (mean ± SD; *n* = 11).

Characteristic	Measure
No. female (%)	6 (55)
Age [mean (range), years]	35.5 (27–41)
Employed as a taxi driver (years)	6.0 ± 3.4
BMI (kg/m^2^)	26.1 ± 3.5
Seated blood pressure	
Systolic (mm Hg)	107.7 ± 11.3
Diastolic (mm Hg)	71.8 ± 9.6
Resting mean heart rate (beats/min)	63.4 ± 6.2
Cholesterol (mg/dL)	173.2 ± 29.4
Triglycerides (mg/dL)	94.6 ± 45.9
High-density lipoprotein (mg/dL)	53.3 ± 8.3
Low-density lipoprotein (mg/dL)	94.0 ± 17.3

**Table 2:** Daily averages of exposure variables inside the taxicab.

	Period^a^			Percentile^b^				
Variable	Before	During	After	25	50	75	95	IQR
PM_2.5_, real time (μg/m^3^)	95.4 ± 58.6	39.5 ± 25.2	64.0 ± 80.3	22.7	44.6	84.8	207.2	62.1
PM_2.5_, mass (μg/m^3^)	105.5 ± 44.1	45.2 ± 27.0	80.4 ± 72.5	34.6	56.6	104.1	182.5	69.5
CO (ppm)	3.6 ± 1.4	2.8 ± 1.0	2.7 ± 0.7	2.2	2.6	3.7	5.0	1.5
NO_2_ (ppb)	36.4 ± 12.3	30.3 ± 12.2	37.1 ± 17.0	24.2	32.8	45.3	61.0	21.1
NO (ppb)	176.1 ± 84.8	156.0 ± 77.2	268.0 ± 55.5	125.6	181.5	274.8	362.2	149.2
Temperature (°C)	30.0 ± 4.4	28.8 ± 2.0	25.0 ± 2.2	24.9	28.4	30.3	33.5	5.4
RH (%)	38.8 ± 9.5	41.7 ± 6.6	24.8 ± 5.8	28.8	35.2	40.9	51.2	12.1

a Before, during, and after the Beijing 2008 Olympic Games indicates the periods of 26 May to 19 June, 11 August to 5 September, and 27 October to 14 November, respectively; concentrations are presented as arithmetic mean ± SD for the three time periods.

b Percentiles were calculated and presented after combining exposure data from all three time periods.

**Table 3:** Distribution of 5-min HRV indices by time period.

		Percentile			
Variable/period	*n*^a^	25	50	75	95
5-min SDNN (msec)					
Before	1,320	34	44	54	78
During	1,366	39	49	58	76
After	1,309	35	44	54	72

5-min LF power (msec^2^)					
Before	1,298	304.7	507.6	830.8	1506.2
During	1,360	353.5	606.2	888.6	1526.2
After	1,287	310.6	500.1	803.1	1509.5

5-min HF power (msec^2^)					
Before	1,298	52.1	97.9	185.1	375.6
During	1,360	81.9	139.0	222.8	420.3
After	1,287	60.9	126.4	228.7	425.6

a Sample size after excluding all abnormities and noises, as well as the data of the time when drivers left the taxicab.

**Table 4:** Estimated percent changes (95% CIs) in 5-min HRV indices in lagged PM_2.5_ moving averages.

	30-min moving average		2-hr moving average	
Variable	All data	Excluding outlying exposure values^a^	All data	Excluding outlying exposure values^a^
5-min SDNN	−2.2 (−3.8 to −0.6)**	−3.5 (−5.7 to −1.2)**	−1.1 (−3.3 to 1.1)	−1.6 (−4.2 to 1.1)
5-min LF power	−2.8 (−6.6 to 1.0)	−5.8 (−10.7 to −0.5)*	−4.2 (−9.0 to 0.8)	−4.8 (−10.6 to 1.3)
5-min HF power	−4.1 (−7.6 to −0.5)*	−7.1 (−11.6 to −2.4)**	−6.2 (−10.7 to −1.5)*	−7.8 (−13.1 to −2.3)**

Effect estimates are percent changes for per IQR (69.5 μg/m^3^) increase of PM_2.5_ moving averages, adjusted for age, time of day, log_10_-transformed heart rate, and linear and quadratic terms of moving averages of real-time temperature/RH corresponding to the PM_2.5_.

a With observations in the top 5% and bottom 5% of the 30-min and 2-hr PM_2.5_ moving averages, respectively, removed.

* *p* < 0.05;

** *p* < 0.01.

## References

[b1-ehp-118-87] Cavallari JM, Eisen EA, Chen JC, Fang SC, Dobson CB, Schwartz J (2007). Night heart rate variability and particulate exposures among boilermaker construction workers. Environ Health Perspect.

[b2-ehp-118-87] Clancy L, Goodman P, Sinclair H, Dockery DW (2002). Effect of air-pollution control on death rates in Dublin, Ireland: an intervention study. Lancet.

[b3-ehp-118-87] Dockery DW (2001). Epidemiologic evidence of cardiovascular effects of particulate air pollution. Environ Health Perspect.

[b4-ehp-118-87] Gold DR, Litonjua A, Schwartz J, Lovett E, Larson A, Nearing B (2000). Ambient pollution and heart rate variability. Circulation.

[b5-ehp-118-87] Goldberg MS, Burnett RT, Yale JF, Valois MF, Brook JR (2006). Associations between ambient air pollution and daily mortality among persons with diabetes and cardiovascular disease. Environ Res.

[b6-ehp-118-87] Holguín F, Téllez-Rojo MM, Hernández M, Cortez M, Chow JC, Watson JG (2003). Air pollution and heart rate variability among the elderly in Mexico City. Epidemiology.

[b7-ehp-118-87] Kang MG, Koh SB, Cha BS, Park JK, Woo JM, Chang SJ (2004). Association between job stress on heart rate variability and metabolic syndrome in shipyard male workers. Yonsei Med J.

[b8-ehp-118-87] La Rovere MT, Pinna GD, Maestri R, Mortara A, Capomolla S, Febo O (2003). Short-term heart rate variability strongly predicts sudden cardiac death in chronic heart failure patients. Circulation.

[b9-ehp-118-87] Larrieu S, Jusot JF, Blanchard M, Prouvost H, Declercq C, Fabre P (2007). Short term effects of air pollution on hospitalizations for cardiovascular diseases in eight French cities: the PSAS program. Sci Total Environ.

[b10-ehp-118-87] Liao D, Creason J, Shy C, Williams R, Watfs R, Zweidinger R (1999). Daily variation of particulate air pollution and poor cardiac autonomic control in the elderly. Environ Health Perspect.

[b11-ehp-118-87] Liao D, Duan Y, Whitsel EA, Zheng ZJ, Heiss G, Chinchilli VM (2004). Association of higher levels of ambient criteria pollutants with impaired cardiac autonomic control: a population-based study. Am J Epidemiol.

[b12-ehp-118-87] Lombardi F, Mäkikallio TH, Myerburg RJ, Huikuri HV (2001). Sudden cardiac death: role of heart rate variability to identify patients at risk. Cardiovasc Res.

[b13-ehp-118-87] Magari SR, Hauser R, Schwartz J, Williams PL, Smith TJ, Christiani DC (2001). Association of heart rate variability with occupational and environmental exposure to particulate air pollution. Circulation.

[b14-ehp-118-87] Magari SR, Schwartz J, Williams PL, Hauser R, Smith TJ, Christiani DC (2002a). The association between personal measurements of environmental exposure to particulates and heart rate variability. Epidemiology.

[b15-ehp-118-87] Magari SR, Schwartz J, Williams PL, Hauser R, Smith TJ, Christiani DC (2002b). The association of particulate air metal concentrations with heart rate variability. Environ Health Perspect.

[b16-ehp-118-87] Park SK, O’Neill MS, Vokonas PS, Sparrow D, Schwartz J (2005). Effects of air pollution on heart rate variability: VA Normative Aging Study. Environ Health Perspect.

[b17-ehp-118-87] Peretz A, Kaufman JD, Trenga CA, Allen J, Carlsten C, Aulet MR (2008). Effects of diesel exhaust inhalation on heart rate variability in human volunteers. Environ Res.

[b18-ehp-118-87] Pope CA, Burnett RT, Thurston GD, Thun MJ, Calle EE, Krewski D (2004a). Cardiovascular mortality and long-term exposure to particulate air pollution: epidemiological evidence of general pathophysiological pathways of disease. Circulation.

[b19-ehp-118-87] Pope CA, Hansen ML, Long RW, Nielsen KR, Eatough NL, Wilson WE (2004b). Ambient particulate air pollution, heart rate variability, and blood markers of inflammation in a panel of elderly subjects. Environ Health Perspect.

[b20-ehp-118-87] Rhoden CR, Wellenius GA, Ghelfi E, Lawrence J, Gonzalez-Flecha B (2005). PM-induced cardiac oxidative stress and dysfunction are mediated by autonomic stimulation. Biochim Biophys Acta.

[b21-ehp-118-87] Riediker M, Cascio WE, Griggs TR, Herbst MC, Bromberg PA, Neas L (2004). Particulate matter exposure in cars is associated with cardiovascular effects in healthy young men. Am J Respir Crit Care Med.

[b22-ehp-118-87] Riojas-Rodriguez H, Esamilla-Cejudo JA, Gonzalez-Hermosillo JA, Tellez-Rojo MM, Vallejo M, Santos-Burgoa C (2006). Personal PM_2.5_ and CO exposures and heart rate variability in subjects with known ischemic heart disease in Mexico City. J Exp Sci Environ Epidemiol.

[b23-ehp-118-87] Saito K, Hiya A, Uemura Y, Furuta M (2008). Clinical training stress and autonomic nervous function in female medical technology students: analysis of heart rate variability and 1/f fluctuation. J Med Invest.

[b24-ehp-118-87] Schwartz J, Litonjua A, Suh H, Verrier M, Zanobetti A, Syring M (2005). Traffic related pollution and heart rate variability in a panel of elderly subjects. Thorax.

[b25-ehp-118-87] Task Force of the European Society of Cardiology and the North American Society of Pacing and Electrophysiology (1996). Heart rate variability: standards of measurement, physiological interpretation, and clinical use. Circulation.

[b26-ehp-118-87] Vallejo M, Ruiz S, Hermosillo AG, Borja-Aburto VH, Cárdenas M (2006). Ambient fine particles modify heart rate variability in young healthy adults. J Exp Sci Environ Epidemiol.

[b27-ehp-118-87] Vrijkotte T, van Doornen L, de Geus E (2000). Effects of work stress on ambulatory blood pressure, heart rate, and heart rate variability. Hypertension.

